# Influenza Vaccination Status and Its Affecting Factors among Stroke Survivors: Findings from the Korea National Health and Nutrition Examination Survey

**DOI:** 10.3390/vaccines9070763

**Published:** 2021-07-08

**Authors:** Eung-Joon Lee, Oh Deog Kwon, Seung Jae Kim

**Affiliations:** 1 Institute of Public Health and Medical Care, Seoul National University Hospital, Seoul 03080, Korea; lejoon0824@gmail.com; 2 Department of Neurology, Seoul National University Hospital, Seoul 03080, Korea; 3 Movinci Clinic, Seoul 06030, Korea; ovirtue@gmail.com; 4 International Healthcare Center, Seoul St. Mary’s Hospital, College of Medicine, The Catholic University of Korea, Seoul 06591, Korea; 5 Department of Family Medicine, Seoul St. Mary’s Hospital, College of Medicine, The Catholic University of Korea, Seoul 06591, Korea

**Keywords:** influenza, vaccination, stroke survivors, associated factors, KNHANES

## Abstract

Few studies have examined the influenza vaccination rates among stroke survivors despite the importance of vaccines in preventing influenza- and stroke-related complications. Thus, we investigated the vaccination rates and the associated factors among stroke survivors using the representative Korea National Health and Nutrition Examination Survey 2014–2018. We measured and compared the vaccination rates of 591 stroke survivors and 17,997 non-stroke survivors. Multivariate logistic regression analyses of all stroke survivors and age subgroups (<65 and ≥65 years) were performed to identify the factors influencing vaccination. The overall vaccination rate was significantly higher in the stroke survivors (64.8%) than in the non-stroke survivors (41.1%), but it was low compared to global standards. Among stroke survivors aged <65 years, the rate was low (37.5%), but it improved in those aged ≥65 years (85.6%). Age ≥ 65 years, the eligible age for the national free vaccination program was the most prominent predictor of vaccination for all stroke survivors, while smoking was a negative predictor. No significant factors were found in the subgroup analyses according to age (<65 and ≥65 years). Therefore, implementing strategic public health policies, such as expanding the free vaccination program to stroke survivors aged <65 years, may improve vaccine coverage.

## 1. Introduction

Influenza is a highly contagious infectious disease that can cause serious complications and even mortality, especially among high-risk populations, such as elderly patients and individuals with chronic conditions [1,2,3]. The number of influenza-associated respiratory deaths worldwide was estimated to be 290,000–650,000 cases in 2018 [4,5]. Previous studies have reported that the annual influenza vaccination is a safe and cost-effective method for preventing influenza and its complications in high-risk groups [6,7,8,9]. Therefore, guidelines for influenza immunization in Korea and other countries strongly recommend high-risk individuals, including those with cardiovascular diseases, to receive seasonal influenza vaccines [10,11]. Patients with a history of stroke, a major atherosclerotic vascular disease, are also required to receive an annual influenza vaccination as they are more vulnerable to influenza-related complications, including death [6,7,12]. Furthermore, previous studies have reported that influenza infection also directly triggers stroke morbidity and stroke-related mortality [13,14], which could be prevented by influenza vaccination [15,16,17,18,19]. The American Heart Association and the American College of Cardiology recommend that patients with coronary and other atherosclerotic vascular diseases should receive influenza vaccination for secondary prevention [20]. Nevertheless, very few studies have examined the coverage rates of influenza vaccination and its affecting factors among stroke survivors. Some nationwide studies conducted in the United States and Korea investigated the influenza vaccination status of patients with cardiovascular diseases, including stroke survivors, and found suboptimal coverage rates in both countries [21,22]. However, these studies did not distinguish the coverage rates of stroke survivors alone, and the results were relatively outdated. One study in the United States reported poor influenza vaccination coverage among stroke survivors, but the results were outdated [23]. Thus, this study aimed to investigate the influenza vaccination coverage rates among Korean patients with stroke and its influencing factors using more recent nationally representative survey data.

## 2. Methods

### 2.1. Study Population and Data Collection

**Figure 1:**
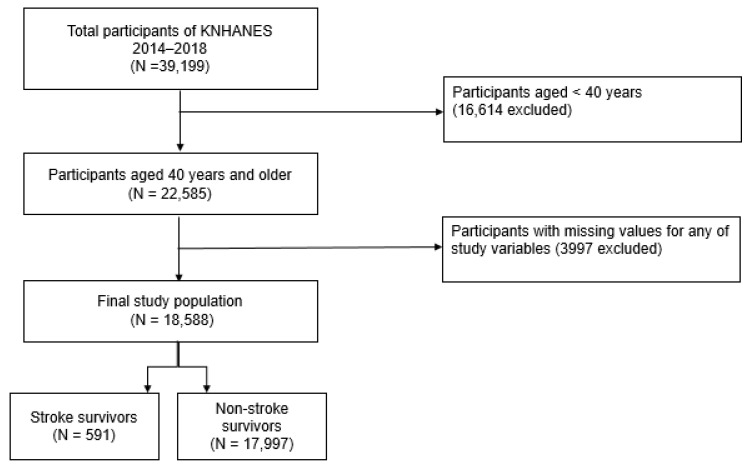
Flow diagram of selection of study population. KNHANES, Korea National Health and Nutrition Examination Survey.

The present study was conducted using data from the Korea National Health and Nutrition Survey (KNHANES) between 2014 and 2018. The KNHANES, a nationwide cross-sectional survey examining the non-institutionalized civilian Korean population, has been performed annually by the Korea Centers for Disease Control and Prevention (KCDC) since 1998. It offers extensive information on individuals’ sociodemographics, health status, and health behaviors. The KNHANES participants are selected through complex, stratified, multi-stage, and probability sampling to provide nationally representative and non-biased data. Further information regarding the validity and representativeness of the KNHANES has been documented in previous studies [24,25]. Among the 39,199 total participants of the KNHANES 2014–2018, those aged less than 40 years were excluded (N = 16,614). Participants with missing data on any of the study variables, including those who did not respond to questions about the status of influenza vaccination and previous diagnosis of stroke, were also excluded (N = 3997). Subsequently, 18,588 participants were selected as the study population. Participants were then dichotomized into those with (N = 591) and without (N = 17,997) a history of stroke (Figure 1). Participants were considered stroke survivors if they responded positively to a prior diagnosis of stroke by a physician. The Institutional Review Board of the Seoul St. Mary’s Hospital, Catholic University of Korea (approval number: KC21ZASI0138) approved this study, and the requirement for written informed consent was waived.

### 2.2. Definition of Influenza Vaccination Status

Influenza vaccination coverage rates were assessed based on the self-reported influenza vaccination status within a year. Since 2005, the KCDC has provided free influenza vaccination to elderly adults aged ≥65 years through the national immunization program at public health centers [26]. 

### 2.3. Factors Associated with Influenza Vaccination Status

The potential predictors of receiving an influenza vaccine were classified into three broad groups based on the self-administered questionnaire of the KNHANES—sociodemographic, health status, and health behavior factors. The sociodemographic factors included age, sex, marital status, educational status, employment status, household income, residential area, and type of health insurance. The health status factors included body mass index (BMI), comorbidities, self-rated health status, and limitations in daily activities. Lastly, the health behavior factors included smoking status, alcohol status, degree of physical activity, and regular health checkups. BMI was calculated by dividing the participant’s weight in kilograms with their height in squared meters. Obesity was defined based on the cutoff BMI values in Korea (underweight: <18.5 kg/m^2^, normal weight: 18.5–22.9 kg/m^2^, overweight: 23–24.9 kg/m^2^, and obese: ≥25.0 kg/m^2^) [27]. We included patients with hypertension, diabetes mellitus (DM), ischemic heart disease (myocardial infarction and angina), cancers (stomach, liver, breast, lung, thyroid, and other cancers), chronic pulmonary diseases (chronic obstructive pulmonary disease and asthma), liver cirrhosis, and chronic renal disease. The definitions of each condition were based on a physician’s self-reported prior diagnosis. Smoking status was categorized as a current smoker or non-smoker (never or past smoker). Risky drinking was defined as drinking at least twice a week with an average of seven standard drinks or more per occasion for male participants and five standard drinks or more per occasion for female participants. Participants who were non-drinkers or drank less than the criteria for risky drinking were considered non-risky drinkers. Physical activity was assessed based on the Physical Activity Guidelines for Americans, 2nd edition [28]. These guidelines state that adults need to perform at least 150 min of moderate-intensity aerobic physical activity or at least 75 min of vigorous-intensity aerobic physical activity per week or an equivalent combination of moderate- and vigorous-intensity activity to gain health benefits [28]. When the participant’s level of physical activity met these criteria, they were considered to have “sufficient physical activity.” Lastly, regular health screening was defined as a positive response to whether the participant underwent health screening within the last two years. 

### 2.4. Statistical Analysis

The sampling weights provided by the KCDC were used for all statistical analyses to ensure unbiased nationally representative estimates [24]. The baseline characteristics of stroke and non-stroke survivors, analyzed through descriptive statistics, are presented as percentages and standard errors or as means and standard deviations. The differences in the characteristics of the groups were compared using the chi-square test for proportions and the adjusted Wald test for means. We calculated the overall influenza vaccination coverage rates and compared them between stroke and non-stroke survivors according to their baseline characteristics. The coverage rates between the two groups were compared using the adjusted Wald test. Then, using the above mentioned factors as covariates, we conducted univariate and multivariate logistic regression analyses to identify the factors that affect influenza vaccination status among all stroke survivors and age subgroups (elderly (aged ≥65 years) vs. non-elderly (aged <65 years) stroke survivors). Factors with *P*-values less than 0.1 in univariate analyses were included in multivariate analysis. All statistical analyses were performed using STATA version 14.1 (Stata Corp., College Station, TX, USA), and *P*-values less than 0.05 were considered statistically significant. 

## 3. Results

### 3.1. Characteristics of the Participants with and without Stroke

The baseline characteristics of the stroke and non-stroke survivors are summarized in Table 1. In total, 55.5% and 52.1% of participants were men among stroke survivors and non-stroke survivors, respectively. The mean age of the stroke survivors was 65.9 ± 0.5 years, and 56.7% of stroke survivors were aged 65 years and older. In contrast, the non-stroke survivors were younger (mean age: 56.1 ± 0.1 years), and 23.0% of non-stroke survivors were aged 65 years and older. These differences were statistically significant. In addition, stroke survivors were more likely to be single/divorced/separated/widowed (31.6% vs. 19.1%), unemployed (65.0% vs. 35.0%), be medical aid beneficiaries (13.0% vs. 4.1%), reside in rural areas (41.3% vs. 36.9%), have lower education, and have less income (lower-middle or lower class: 68.1% vs. 41.5%) than non-stroke survivors. They also had higher BMI (mean BMI 24.6 ± 0.1 kg/m^2^ vs. 24.1 ± 0.0 kg/m^2^), poorer self-rated health status (very poor/poor self-rated health: 50.7% vs. 19.5%), more comorbidities (74.5% vs. 36.7%), and lower frequency of undergoing health screening within the last 2 years than non-stroke survivors. In terms of the specific comorbidities, stroke survivors were more likely to have hypertension (68.1% vs. 26.8%), DM (30.0% vs. 10.3%), and ischemic heart disease (12.9% vs. 2.8%) than non-stroke survivors. The differences in the other characteristics between stroke and non-stroke survivors were insignificant. 

### 3.2. Comparison of Influenza Vaccination Status between the Stroke and Non-Stroke Survivors

The overall influenza vaccination coverage rate for stroke survivors (64.8%) was significantly higher than that for non-stroke survivors (41.1%). Subgroup analysis according to baseline characteristics also demonstrated that influenza vaccination rates were all significantly higher in stroke survivors than in non-stroke survivors, except in those aged 65 years and older, with college or higher degrees, high income, DM, and chronic renal disease and those who were underweight and medical aid beneficiaries (Table 2).

### 3.3. Predictors of Influenza Vaccination Coverage among the Stroke Survivors

Table 3 presents the results of univariate and multivariate logistic regression analyses for the factors associated with influenza vaccination coverage among stroke survivors. In univariate analyses, age ≥ 65 years (odds ratio (OR) 9.90, 95% confidence interval (CI) 6.21–15.78) was the most prominent predictor of receiving an influenza vaccine among stroke survivors. Female sex (OR 2.01, 95% CI 1.33–3.04) and comorbidities (OR 1.86, 95% CI 1.17–2.95) were also positively associated with receiving an influenza vaccine. In contrast, employment (OR 0.32, 95% CI 0.21–0.49), higher education (OR 0.44, 95% CI 0.33–0.59), higher income (OR 0.69, 95% CI 0.56–0.85), smoking (OR 0.35, 95% CI 0.21–0.59), risky drinking (OR 0.44, 95% CI 0.21–0.96), and sufficient physical activity (OR 0.49, 95% CI 0.31–0.76) were negatively associated with receiving an influenza vaccine. In multivariate analysis, age ≥ 65 years (adjusted OR (aOR) 7.51, 95% CI 4.42–12.75) was consistently identified as the most significant determinant of receiving an influenza vaccine, while smoking (aOR 0.44, 95% CI 0.24–0.81) was consistently demonstrated to have a negative correlation with receiving an influenza vaccine. However, other factors that demonstrated significant associations with influenza vaccination in univariate analyses were not significant in multivariate analysis. The results of the logistic regression analyses for stroke survivors aged <65 years and aged ≥65 years are presented in Table 4 and Table 5, respectively. For stroke survivors aged <65 years, female sex (OR 2.03, 95% CI 1.04–3.95) and older age (OR 1.09, 95% CI 1.02–1.16) were identified as positive predictors for receiving an influenza vaccine, while employment (OR 0.49, 95% CI 0.25–0.98), higher education (OR 0.45, 95% CI 0.29–0.70), better self-rated health status (OR 0.60, 95% CI 0.37–1.00), smoking (OR 0.37, 95% CI 0.15–0.88), and sufficient physical activity (OR 0.42, 95% CI 0.21–0.86) were negative predictors in univariate analyses. However, none of these factors were significantly associated with influenza vaccination in multivariate analysis. For stroke survivors aged ≥65 years, only older age and better self-rated health status were included as covariates in multivariate analysis as these were the only variables that had *P*-values less than 0.1 in univariate analyses. However, these factors did not show any significant correlations with influenza vaccination. 

## 4. Discussion

Our study demonstrated that the overall influenza vaccination coverage rate was significantly higher among stroke survivors than among non-stroke survivors in Korea between 2014 and 2018. This result was likely because stroke survivors are more health-conscious than non-stroke survivors. According to the Health Belief Model, individuals who perceive their health issues as more serious would likely engage in healthier behaviors to prevent worse outcomes [29]. Thus, the medical history of stroke may have prompted the stroke survivors to receive influenza vaccines more actively. Other Korean studies have also found that the influenza vaccination coverage rates in patients with chronic diseases, such as DM, cancers, and cardiovascular diseases, were considerably higher than in those without these conditions [22,30,31]. However, it seems that the Health Belief Model was insufficient in encouraging stroke survivors to receive the influenza vaccine as the overall coverage rate of 64.8% falls considerably short of the global target coverage rate for high-risk groups. The United States has set a goal of 90% influenza vaccination coverage rate for high-risk patients in the Healthy People 2020 document, while the European Union Council has proposed a target vaccination rate of 75% [32,33]. The numbers are far more concerning for stroke survivors aged <65 years, who have a coverage rate of 37.5%. Given that the influenza vaccine prevents influenza infection and its related complications [6,7] and protects patients from stroke and its related mortality [15,16,17,18,19], more attention and efforts are needed to improve the coverage rate of stroke survivors, especially those aged <65 years. 

For the factors associated with receiving an influenza vaccine, age ≥65 years was the most prominent positive predictor among the stroke survivors; no significant association was found between other factors, except smoking, and receiving a vaccine. This trend is believed to be attributed mainly to KCDC’s national immunization program, which offers free influenza vaccination to every adult aged ≥65 years [26]. Owing to this program, Korea has significantly higher influenza vaccination coverage rates among the elderly aged ≥65 years (>80%) than the other member countries of the Organization for Economic Cooperation and Development [34,35,36]. In contrast, the coverage is comparatively low (25–30%) among younger adults aged <65 years in Korea as they are not eligible for free vaccination [34,35]. Previous Korean studies have confirmed that the coverage rate of patients aged <65 years with chronic diseases (e.g., DM and cardiovascular disease) was slightly higher than those aged <65 years without chronic diseases since those with chronic diseases are likely to be more health conscious [22,30]. However, owing to the free vaccination program, significant disparities are present in the coverage rates of patients with the said chronic diseases aged <65 years and those aged ≥65 years [22,30]. Both trends were consistently observed in the coverage rates of stroke survivors in the present study. Further, we believe that the free vaccination program for the elderly population is the reason for the insignificant difference in the coverage rates between stroke and non-stroke survivors aged ≥65 years and the significant difference in the rates between stroke and non-stroke survivors aged <65 years. 

In contrast, smoking was significantly associated with a lower vaccination rate in this study. Smokers are more susceptible to influenza, which can also be fatal [37]. In addition, persistent smoking after stroke is highly associated with stroke recurrence [38]. Thus, the importance of receiving influenza vaccination should be especially emphasized to stroke survivors who are currently smoking. 

We additionally performed multivariate subgroup analyses according to age (<65 and ≥65 years), but no significant factors that were predictive of receiving an influenza vaccine for both subgroups were noted. This may imply that offering free vaccinations is the most critical factor that motivates stroke survivors to be vaccinated. Furthermore, their vaccination behavior tends to be unaffected by their other characteristics, including sociodemographics, health status, and health behaviors. Previous studies have confirmed that vaccination recommendation alone is not sufficient to promote higher coverage and that implementing public health policies that reduce patients’ vaccination costs has a more powerful impact on increasing coverage rates [39,40]. Thus, implementing strategic public health policies, such as expanding the eligibility of free vaccination candidates by setting a cutoff age lower than the current age of 65, could be a way to encourage more stroke survivors aged <65 years to get vaccinated against influenza. Further studies regarding the factors influencing influenza vaccination among stroke survivors and the cost-effectiveness of expanding free vaccination programs to younger stroke survivors are needed to verify our findings and to improve stroke survivors’ coverage rates aged <65 years. 

The major strength of our study is that we objectively analyzed the recent influenza vaccination status among stroke survivors by accurately reflecting the entire population of Korea using a nationally representative survey database. Despite its importance, very few studies have investigated the influenza vaccination coverage rate among stroke survivors, especially in Korea. Second, we examined the factors influencing influenza vaccination behavior of stroke survivors by including various aspects of their characteristics (such as sociodemographic, health status, and health behavior factors) in the analyses to offer an essential public health perspective for improving the vaccination coverage of stroke survivors. 

However, this study has several limitations. First, potential underestimation of vaccination coverage rates may have been possible since the KNHANES is conducted throughout the year, whereas influenza vaccinations are generally conducted before the annual epidemic season (usually from September to December). Thus, recall bias could have occurred if a time gap existed between the survey and vaccination periods. Second, reporting bias could not be ruled out in other study variables as the KNHANES data were obtained through a self-administered questionnaire. Third, other factors that could affect the vaccination behavior, such as stroke severity, prior history of influenza infection, participant’s belief in the vaccine’s effectiveness, and knowledge of vaccination recommendations, could not be assessed because these were not included in the KNHANES. 

## 5. Conclusions

Despite being strongly recommended by various practice guidelines, the influenza vaccination coverage rate among stroke survivors was low in Korea (64.8%). The rate among participants aged <65 years was lower (37.5%) than that among participants aged ≥65 years (85.6%). Age ≥ 65 years, the eligible age for the national free vaccination program, was the most prominent predictor of vaccination against influenza for all stroke survivors, while smoking was a negative predictor. No significant factors were found in subgroup analyses according to age (<65 and ≥65 years). Therefore, implementing strategic public health policies, such as expanding the free vaccination program to stroke survivors aged <65 years, could be considered to improve coverage rates.

## Figures and Tables

**Table 1:** Baseline characteristics of those with and without stroke.

Characteristics	Stroke (N = 591) % (SE) or Mean ± SD	Non-Stroke (N = 17,997) % (SE) or Mean ± SD	*p*-Value
Sociodemographic factors			
Sex			0.002
Male	55.5 (2.4)	52.1 (0.3)	
Female	44.5 (2.4)	47.8 (0.3)	
Age (years)	65.9 ± 0.5	56.1 ± 0.1	0.000
40–64	43.3 (2.5)	77.0 (0.5)	0.000
≥65	56.7 (2.5)	23.0 (0.5)	0.000
Marital status			0.000
Married	68.4 (2.3)	80.9 (0.5)	
Single/divorced/separated/widowed	31.6 (2.3)	19.1 (0.5)	
Employment status			0.000
Employed	35.0 (2.5)	65.0 (0.5)	
Unemployed	65.0 (2.5)	35.0 (0.5)	
Education status			0.000
Middle school or lower	62.0 (2.4)	32.4 (0.6)	
High school	25.3 (2.1)	34.0 (0.5)	
College or higher	12.7 (1.6)	33.6 (0.7)	
Household Income			0.000
Low	42.7 (2.4)	17.9 (0.5)	
Lower middle	25.4 (2.1)	23.6 (0.5)	
Upper middle	18.8 (2.0)	27.6 (0.5)	
High	13.1 (1.8)	30.9 (0.8)	
Residential area			0.091
Urban	58.7 (2.7)	63.1 (0.9)	
Rural	41.3 (2.7)	36.9 (0.9)	
Health insurance			0.000
National health insurance	87.0 (1.7)	95.9 (0.2)	
Medicaid	13.0 (1.7)	4.1 (0.2)	
Health status factors			
BMI (kg/m^2^)	24.6 ± 0.1	24.1 ± 0.0	0.002
<18.5	1.6 (0.5)	2.4 (0.1)	0.062
18.5–22.9	30.5 (2.4)	36.0 (0.4)	0.062
23–24.9	26.8 (2.3)	25.1 (0.4)	0.062
≥25	41.1 (2.4)	36.5 (0.4)	0.062
Comorbidities			0.000
No	24.5 (2.1)	63.3 (0.5)	
Yes	74.5 (2.1)	36.7 (0.5)	
Hypertension	68.1 (2.4)	26.8 (0.4)	0.000
Diabetes mellitus	30.0 (2.2)	10.3 (0.3)	0.000
Ischemic heart disease *	12.9 (1.6)	2.8 (0.1)	0.000
Cancer ^†^	6.1 (1.2)	6.0 (0.2)	0.871
Chronic pulmonary diseases ^‡^	4.4 (1.0)	3.1 (0.1)	0.106
Liver cirrhosis	0.3 (0.2)	0.5 (0.1)	0.488
Chronic renal disease	0.6 (0.4)	0.4 (0.1)	0.428
Self-rated health			0.000
Very poor/poor	50.7 (2.5)	19.5 (0.4)	
Fair	39.7 (2.4)	52.9 (0.4)	
Good/excellent	9.6 (1.5)	27.6 (0.4)	
Limitation in daily activities			0.000
Yes	30.7 (2.4)	8.4 (0.3)	
No	69.3 (2.4)	91.6 (0.3)	
Health behavior factors			
Smoking status			0.696
Never/past smoker	81.2 (1.9)	80.4 (0.4)	
Current smoker	18.8 (1.9)	19.6 (0.4)	
Drinking status			0.114
Non-risky drinking	90.6 (1.6)	87.6 (0.3)	
Risky drinking	9.4 (1.6)	12.4 (0.3)	
Physical activity			0.003
Sufficient	35.6 (2.4)	44.5 (0.5)	
Insufficient	64.4 (2.4)	55.5 (0.5)	
Health check-up within the last 2 years			0.000
Yes	64.9 (2.2)	74.1 (0.4)	
No	35.1 (2.2)	25.9 (0.4)	

All data were weighted to the standard Korean population. *P*-values were obtained by chi-square test for proportions or adjusted Wald test for means. * Myocardial infarction or angina pectoris. ^†^ Stomach, liver, colon, breast, cervix, lung, thyroid cancer, and other cancers. ^‡^ Asthma and chronic obstructive pulmonary disease. Abbreviation: SE, standard error; SD, standard deviation; BMI, body mass index.

**Table 2:** Comparison of influenza vaccination coverage between stroke and non-stroke survivors.

	Stroke (N = 591)	Non-Stroke (N = 17,997)	
Variables	Vaccinated %(SE)	Vaccinated %(SE)	*p*-Value
Overall	64.8 (2.4)	41.1 (0.5)	0.000
Sociodemographic factors			
Sex			
Male	57.8 (3.4)	35.6 (0.6)	0.000
Female	73.4 (3.1)	46.2 (0.6)	0.000
Age (years)			
40–64	37.5 (4.0)	28.7 (0.5)	0.030
≥65	85.6 (2.0)	82.6 (0.6)	0.152
Marital status			
Married	63.8 (3.0)	38.6 (0.6)	0.000
Single/divorced/separated/widowed	66.8 (4.2)	51.6 (1.0)	0.000
Employment status			
Employed	47.8 (4.1)	33.6 (0.6)	0.000
Unemployed	73.9 (2.6)	55.0 (0.8)	0.001
Educational status			
Middle school or lower	75.3 (2.6)	61.3 (0.8)	0.000
High school	51.1 (5.2)	33.9 (0.8)	0.001
College or higher	40.3 (6.8)	28.9 (0.8)	0.098
Household Income			
Low	74.1 (3.5)	63.1 (1.0)	0.003
Lower middle	61.1 (4.7)	41.9 (1.0)	0.000
Upper middle	61.8 (5.7)	34.3 (0.9)	0.000
High	45.5 (7.5)	33.8 (0.8)	0.117
Residential area			
Urban	66.8 (3.2)	40.6 (0.6)	0.000
Rural	61.9 (3.6)	41.9 (0.9)	0.000
Health insurance			
National health insurance	64.9 (2.6)	40.5 (0.5)	0.000
Medical aid	63.5 (6.2)	54.8 (2.1)	0.173
Health status factors			
BMI (kg/m^2^)			
<18.5	53.6 (17.5)	39.7 (2.5)	0.434
18.5–22.9	62.7 (4.6)	40.1 (0.7)	0.000
23–24.9	61.4 (4.8)	41.8 (0.9)	0.000
≥25	68.9 (3.6)	41.7 (0.8)	0.000
Comorbidities			
No	68.3 (2.7)	32.3 (0.6)	0.000
Yes	53.8 (5.1)	56.3 (0.8)	0.000
Hypertension	68.6 (2.7)	58.7 (0.9)	0.000
Diabetes mellitus	66.2 (4.4)	60.4 (1.4)	0.207
Ischemic heart disease *	68.3 (6.3)	67.9 (2.2)	0.000
Cancer ^†^	92.4 (3.9)	57.1 (1.7)	0.000
Chronic pulmonary diseases ^‡^	68.6 (11.3)	60.8 (2.5)	0.496
Liver cirrhosis	100.0 (0.0)	45.6 (6.2)	0.000
Chronic renal disease	49.9 (28.2)	68.4 (6.2)	0.523
Self-rated health			
Very poor/poor	66.1 (3.3)	52.8 (1.0)	0.000
Fair	61.6 (3.7)	39.5 (0.6)	0.000
Good/excellent	71.0 (7.4)	35.9 (0.8)	0.000
Limitation in daily activities			
Yes	64.8 (4.4)	58.0 (1.5)	0.138
No	64.8 (2.8)	39.5 (0.5)	0.000
Health behavior factors			
Smoking status			
Never/past smoker	69.5 (2.5)	44.5 (0.6)	0.000
Current smoker	44.4 (5.9)	26.9 (0.9)	0.004
Drinking status			
Non-risky drinking	66.6 (2.4)	43.3 (0.5)	0.000
Risky drinking	47.0 (9.5)	25.5 (1.2)	0.027
Physical activity			
Sufficient	53.9 (4.5)	38.5 (0.7)	0.001
Insufficient	70.6 (2.7)	43.0 (0.6)	0.000
Health check-up within the last 2 years			
Yes	67.1 (2.9)	43.0 (0.6)	0.000
No	60.5 (4.2)	35.7 (0.9)	0.000

All data were weighted to the standard Korean population. * Myocardial infarction or angina pectoris. ^†^ Stomach, liver, colon, breast, cervix, lung, thyroid cancer, and other cancers. ^‡^ Asthma and chronic obstructive pulmonary disease. Abbreviation: SE, standard error; BMI, body mass index.

**Table 3:** Factors associated with influenza vaccination status among stroke survivors (n = 591).

	Univariate Logistic Regression Analysis		Multivariate Logistic Regression Analysis	
Variables	Crude OR (95% CI)	*p*-Value	Adjusted OR * (95% CI)	*p*-Value
Sociodemographic factors				
Female sex	2.01 (1.33–3.04)	0.001	1.29 (0.77–2.17)	0.325
Age (years)				
40–64	Reference		Reference	
≥65	9.90 (6.21–15.78)	0.000	7.51 (4.42–12.75)	0.000
Married	0.87 (0.56–1.37)	0.560		
Employed	0.32 (0.21–0.49)	0.000	0.78 (0.47–1.28)	0.316
Higher education	0.44 (0.33–0.59)	0.000	0.76 (0.55–1.05)	0.096
Higher income	0.69 (0.56–0.85)	0.001	0.94 (0.74–1.20)	0.632
Urban residency	1.24 (0.82–1.87)	0.304		
National Health insurance	1.06 (0.61–1.87)	0.827		
Health status factors				
Higher BMI	1.03 (0.97–1.09)	0.371		
Comorbidities ^†^	1.86 (1.17–2.95)	0.009	1.59 (0.91–2.77)	0.104
Better self-rated health	0.99 (0.74–1.33)	0.956		
Limited daily activities	1.00 (0.65–1.55)	0.996		
Health behavior factors				
Smoking	0.35 (0.21–0.59)	0.000	0.44 (0.24–0.81)	0.009
Risky drinking	0.44 (0.21–0.96)	0.040	1.78 (0.67–4.67)	0.245
Sufficient physical activity	0.49 (0.31–0.76)	0.002	0.69 (0.42–1.13)	0.137
Regular health check-ups ^‡^	1.33 (0.86–2.06)	0.196		

All data were weighted to the standard Korean population. Analyses were performed by univariate and multivariate logistic regression models. * Adjusted for age, sex, education status, employment status, household income, comorbidities, smoking status, drinking status, and degree of physical activity. ^†^ Hypertension, diabetes mellitus, cancer, chronic pulmonary diseases, liver cirrhosis, and chronic renal disease. ^‡^ Receiving health check-ups within the last two years. Abbreviation: CVD, cardiovascular disease; OR, odds ratio; CI, confidence interval; BMI, body mass index.

**Table 4:** Factors associated with influenza vaccination status among stroke survivors aged less than 65 years (n = 199).

	Univariate Logistic Regression Analysis		Multivariate Logistic Regression Analysis	
Variables	Crude OR (95% CI)	*p*-Value	Adjusted OR * (95% CI)	*p*-Value
Sociodemographic factors				
Female sex	2.03 (1.04–3.95)	0.037	1.53 (0.73–3.23)	0.260
Older age	1.09 (1.02–1.16)	0.015	1.07 (0.99–1.17)	0.102
Married	1.48 (0.74–2.97)	0.265		
Employed	0.49 (0.25–0.98)	0.045	0.76 (0.36–1.60)	0.474
Higher education	0.45 (0.29–0.70)	0.000	0.63 (0.39–1.03)	0.065
Higher income	0.81 (0.60–1.10)	0.181		
Urban residency	1.53 (0.78–3.00)	0.216		
National health insurance	0.77 (0.33–1.81)	0.554		
Health status factors				
Higher BMI	1.03 (0.94–1.13)	0.560		
Comorbidities ^†^	1.43 (0.69–2.94)	0.335		
Better self-rated health	0.60 (0.37–1.00)	0.049	0.72 (0.42–1.22)	0.221
Limited daily activities	0.97 (0.46–2.03)	0.933		
Health behavior factors				
Smoking	0.37 (0.15–0.88)	0.024	0.42 (0.14–1.20)	0.104
Risky drinking	1.36 (0.53–3.48)	0.523		
Sufficient physical activity	0.42 (0.21–0.86)	0.018	0.53 (0.23–1.15)	0.109
Regular health check-ups ^‡^	1.60 (0.80–3.20)	0.180		

All data were weighted to the standard Korean population. Analyses were performed by univariate and multivariate logistic regression models. * Adjusted for age, sex, education status, employment status, level of self-rated health, smoking status, and degree of physical activity status. ^†^ Hypertension, diabetes mellitus, cancer, chronic pulmonary diseases, liver cirrhosis, and chronic renal disease. ^‡^ Receiving health check-ups within the last two years. Abbreviation: CVD, cardiovascular disease; OR, odds ratio; CI, confidence interval; BMI, body mass index.

**Table 5:** Factors associated with influenza vaccination status among stroke survivors aged 65 years and older (n = 392).

	Univariate Logistic Regression Analysis		Multivariate Logistic Regression Analysis	
Variables	Crude OR (95% CI)	*p*-Value	Adjusted OR * (95% CI)	*p*-Value
Sociodemographic factors				
Female sex	1.23 (0.63–2.39)	0.547		
Older age	1.07 (1.00–1.15)	0.064	1.07 (0.99–1.14)	0.076
Married	1.11 (0.53–2.32)	0.783		
Employed	0.90 (0.45–1.82)	0.774		
Higher education	1.33 (0.76–2.31)	0.319		
Higher income	0.96 (0.68–1.35)	0.794		
Urban residency	1.34 (0.71–2.51)	0.362		
National health insurance	1.68 (0.74–3.80)	0.216		
Health status factors				
Higher BMI	1.00 (0.91–1.10)	0.939		
Comorbidities ^†^	1.45 (0.60–3.49)	0.408		
Better self-rated health	1.52 (1.01–2.28)	0.046	1.48 (0.99–2.22)	0.059
Limited daily activities	0.89 (0.46–1.70)	0.718		
Health behavior factors				
Smoking	0.65 (0.29–1.46)	0.296		
Risky drinking	0.29 (0.06–1.49)	0.140		
Sufficient physical activity	1.11 (0.57–2.15)	0.769		
Regular health check-ups ^‡^	1.27 (0.67–2.31)	0.454		

All data were weighted to the standard Korean population. Analyses were performed by univariate and multivariate logistic regression models. * Adjusted for age and level of self-rated health. ^†^ Hypertension, diabetes mellitus, cancer, chronic pulmonary diseases, liver cirrhosis, and chronic renal disease. ^‡^ Receiving health check-ups within the last two years. Abbreviation: CVD, cardiovascular disease; OR, odds ratio; CI, confidence interval; BMI, body mass index.

## Data Availability

The data used in this study were obtained from Korea National Health and Nutrition Examination Survey (KNHANES). These data can be downloaded from the following website: https://knhanes.kdca.go.kr/knhanes/main.do.

## References

[1] Atkinson W., Wolfe S., Hamborsky J. (2011). Control CfD, Prevention. Epidemiology and Prevention of Vaccine Preventable Diseases.

[2] Sprenger M.J., Mulder P.G., Beyer W.E., Van Strik R., Masurel N. (1993). Impact of influenza on mortality in relation to age and underlying disease, 1967–1989. Int. J. Epidemiol..

[3] Kwon D.S., Kim K., Park S.M. (2016). Factors associated with influenza vaccination coverage among the elderly in South Korea: The Fourth Korean National Health and Nutrition Examination Survey (KNHANES IV). BMJ Open.

[4] Paget J., Spreeuwenberg P., Charu V., Taylor R.J., Iuliano A.D., Bresee J., Simonsen L., Viboud C., Global Seasonal Influenza-Associated Mortality Collaborator Network and GLaMOR Collaborating Teams (2019). Global mortality associated with seasonal influenza epidemics: New burden estimates and predictors from the GLaMOR Project. J. Glob. Health.

[5] Iuliano A.D., Roguski K.M., Chang H.H., Muscatello D.J., Palekar R., Tempia S., Cohen C., Gran J.M., Schanzer D., Cowling B.J. (2018). Estimates of global seasonal influenza-associated respiratory mortality: A modelling study. Lancet.

[6] Hak E., Buskens E., van Essen G.A., de Bakker D.H., Grobbee D.E., Tacken M.A., van Hout B.A., Verheij T.J. (2005). Clinical effectiveness of influenza vaccination in persons younger than 65 years with high-risk medical conditions: The PRISMA study. Arch. Intern. Med..

[7] Nichol K.L., Wuorenma J., Von Sternberg T. (1998). Benefits of influenza vaccination for low-, intermediate-, and high-risk senior citizens. Arch. Intern. Med..

[8] Govaert T.M., Thijs C., Masurel N., Sprenger M., Dinant G., Knottnerus J. (1994). The efficacy of influenza vaccination in elderly individuals: A randomized double-blind placebo-controlled trial. JAMA.

[9] Nichol K.L., Margolis K., Wuorenma J., Von Sternberg T. (1994). The efficacy and cost effectiveness of vaccination against influenza among elderly persons living in the community. N. Engl. J. Med..

[10] Baek J.H., Seo Y.B., Choi W.S., Kee S.Y., Jeong H.W., Lee H.Y., Eun B.W., Choo E.J., Lee J., Kim S.R. (2014). Guideline on the prevention and control of seasonal influenza in healthcare setting. Korean J. Intern. Med..

[11] Grohskopf L.A., Alyanak E., Broder K.R., Blanton L.H., Fry A.M., Jernigan D.B., Atmar R.L. (2020). Prevention and control of seasonal influenza with vaccines: Recommendations of the Advisory Committee on Immunization Practices—United States, 2020–2021 influenza season. MMWR Recomm. Rep..

[12] Ovbiagele B., McNair N., Pineda S., Liebeskind D.S., Ali L.K., Saver J.L. (2009). A care pathway to boost influenza vaccination rates among inpatients with acute ischemic stroke and transient ischemic attack. J. Stroke Cerebrovasc. Dis..

[13] Nguyen J.L., Yang W., Ito K., Matte T.D., Shaman J., Kinney P.L. (2016). Seasonal influenza infections and cardiovascular disease mortality. JAMA Cardiol..

[14] Muhammad S., Haasbach E., Kotchourko M., Strigli A., Krenz A., Ridder D.A., Vogel A., Marti H., Al-Abed Y., Planz O. (2011). Influenza Virus Infection Aggravates Stroke Outcome. Stroke.

[15] Nichol K.L., Nordin J., Mullooly J., Lask R., Fillbrandt K., Iwane M. (2003). Influenza vaccination and reduction in hospitalizations for cardiac disease and stroke among the elderly. N. Engl. J. Med..

[16] Grau A.J., Fischer B., Barth C., Ling P., Lichy C., Buggle F.J.S. (2005). Influenza vaccination is associated with a reduced risk of stroke. Stroke.

[17] Chiang M.-H., Wu H.-H., Shih C.-J., Chen Y.-T., Kuo S.-C., Chen T.-L. (2017). Association between influenza vaccination and reduced risks of major adverse cardiovascular events in elderly patients. Am. Heart J..

[18] Lavallée P., Perchaud V., Gautier-Bertrand M., Grabli D., Amarenco P.J.S. (2002). Association between influenza vaccination and reduced risk of brain infarction. Stroke.

[19] Udell J.A., Zawi R., Bhatt D.L., Keshtkar-Jahromi M., Gaughran F., Phrommintikul A., Ciszewski A., Vakili H., Hoffman H.B., Farkouh M.E. (2013). Association between influenza vaccination and cardiovascular outcomes in high-risk patients: A meta-analysis. JAMA.

[20] Davis M.M., Taubert K., Benin A.L., Brown D.W., Mensah G.A., Baddour L.M., Dunbar S., Krumholz H.M. (2006). Influenza vaccination as secondary prevention for cardiovascular disease: A science advisory from the American Heart Association/American College of Cardiology. J Am Coll Cardiol..

[21] Ajani U.A., Ford E.S., Mokdad A. (2005). H. Examining the coverage of influenza vaccination among people with cardiovascular disease in the United States. Am. Heart J..

[22] Kim E.Y., Ko J.H., Kim Y.S., Oh P.C. (2020). Prevalence and associated factors of influenza vaccination coverage in Korean adults with cardiovascular disease. Medicine.

[23] Sanossian N., Gatto N.M., Ovbiagele B. (2009). Patterns of influenza vaccination among stroke survivors. Neuroepidemiology.

[24] Kweon S., Kim Y., Jang M.-J., Kim Y., Kim K., Choi S., Chun C., Khang Y., Oh K. (2014). Data resource profile: The Korea national health and nutrition examination survey (KNHANES). Int. J. Epidemiol..

[25] Kim Y. (2014). The Korea National Health and nutrition examination survey (KNHANES): Current status and challenges. Epidemiol. Health.

[26] Yun J.-W., Noh J.Y., Song J.Y., Chun C., Kim Y., Cheong H.J. (2017). The Korean influenza national immunization program: History and present status. Infect. Chemother..

[27] Oh S.W. (2011). Obesity and metabolic syndrome in Korea. Diabetes Metab. J..

[28] Piercy K.L., Troiano R.P., Ballard R.M., Carlson S.A., Fulton J.E., Galuska D.A., George S.M., Olson R.D. (2018). The physical activity guidelines for Americans. JAMA.

[29] Rosenstock I.M., Strecher V.J., Becker M.H. (1988). Social learning theory and the health belief model. Health Educ. Q..

[30] Shin H.-Y., Chung J.H., Hwang H.-J., Kim T.H. (2018). Factors influencing on influenza vaccination and its trends of coverage in patients with diabetes in Korea: A population-based cross-sectional study. Vaccine.

[31] Kim Y.-S., Lee J.-W., Kang H.-T., Kim Y., You H.-S. (2020). Trends in Influenza Vaccination Coverage Rates among Korean Cancer Survivors: Analysis of the Korea National Health and Nutrition Examination Survey III–VI. Korean J. Fam. Med..

[32] Ahlsiö B., Britton M., Murray V., Theorell T. (1984). Disablement and quality of life after stroke. Stroke.

[33] Jorgensen P., Mereckiene J., Cotter S., Johansen K., Tsolova S., Brown C. (2018). How close are countries of the WHO European Region to achieving the goal of vaccinating 75% of key risk groups against influenza? Results from national surveys on seasonal influenza vaccination programmes, 2008/2009 to 2014/2015. Vaccine.

[34] Yang H.J., Cho S.-I. (2014). Influenza vaccination coverage among adults in Korea: 2008–2009 to 2011–2012 seasons. Int. J. Environ. Res. Public Health.

[35] Seo J., Lim J. (2018). Trends in influenza vaccination coverage rates in South Korea from 2005 to 2014: Effect of public health policies on vaccination behavior. Vaccine.

[36] Health at a Glance 2011: OECD Indicators.

[37] Wong C.M., Yang L., Chan K.P., Chan W.M., Song L., Lai H.K., Thach T.Q., Ho L.M., Chan K.H., Lam T.H. (2013). Cigarette smoking as a risk factor for influenza-associated mortality: Evidence from an elderly cohort. Influ. Other Respir. Viruses.

[38] Ovbiagele B., Weir C.J., Saver J.L., Muir K.W., Lees K.R. (2006). Effect of Smoking Status on Outcome after Acute Ischemic Stroke. Cerebrovasc. Dis..

[39] Palache A. (2011). Seasonal influenza vaccine provision in 157 countries (2004–2009) and the potential influence of national public health policies. Vaccine.

[40] Blank P., Schwenkglenks M., Szucs T.D. (2012). The impact of European vaccination policies on seasonal influenza vaccination coverage rates in the elderly. Hum. Vaccines Immunother..

